# A Glimpse of “Dicer Biology” Through the Structural and Functional Perspective

**DOI:** 10.3389/fmolb.2021.643657

**Published:** 2021-05-07

**Authors:** Sneha Paturi, Mandar V. Deshmukh

**Affiliations:** Centre for Cellular and Molecular Biology, Council of Scientific and Industrial Research, Hyderabad, India

**Keywords:** dicer, helicase domain, PAZ domain, RNase III domains, RNAi

## Abstract

The RNA interference pathway (RNAi) is executed by two core enzymes, Dicer and Argonaute, for accomplishing a tailored transcriptional and post-transcriptional gene regulation. Dicer, an RNase III enzyme, initiates the RNAi pathway, plays a pivotal role in fighting infection against pathogens, and acts as a housekeeping enzyme for cellular homeostasis. Here, we review structure-based functional insights of Dicer and its domains present in a diverse group of organisms. Although Dicer and its domains are evolutionarily conserved from microsporidian parasites to humans, recent cryo-electron microscopy structures of *Homo sapiens* Dicer and *Drosophila melanogaster* Dicer-2 suggest characteristic variations in the mechanism of the dsRNA substrate recognition. Interestingly, the necessity for more than one functionally distinct Dicer paralogs in insects and plants compared with a single Dicer in other eukaryotic life forms implies Dicer’s role in the interplay of RNAi and other defense mechanisms. Based on the structural and mechanistic information obtained during the last decade, we aim to highlight the significance of key Dicer domains that are crucial to Dicer specific recognition and precise cleavage of dsRNA substrates. Further, the role of Dicer in the formation of Argonaute-based RNA-induced silencing complex (RISC) assembly formation, Dicer’s ability to regulate a complex protein interaction network, and its role in other cellular processes, as well as its therapeutic potentials, are emphasized.

## Introduction

The defense and counter-defense mechanisms have been shaped by the perpetual arms race between the host and pathogens. The early use of the natural antisense RNA-mediated gene regulation laid the foundations for the development of post-transcriptional gene silencing strategies through the evolution (Tomizawa and Itoh, 1981). The discovery of the first natural antisense RNA in 1981 paved the way for the widespread recognition of various antisense-derived gene regulatory pathways in bacteria and phages (Tomizawa and Itoh, 1981). Subsequently, the evolution of restriction nucleases added more impetus to the already existing antisense-derived defense control. The prokaryotic enzyme pAgo, a homolog of eukaryotic Argonaute (Ago)/P-element Induced Wimpy testis in the *Drosophila melanogaster* (PIWI) family of proteins, cleaves the target mRNA, using a complementary antisense strand derived from a non-coding DNA or RNA (Makarova et al., 2009). Thus, pAgo is functionally analogous to the CRISPR-associated system (CASS) and evolutionarily connected to the eukaryotic RNAi (Makarova et al., 2009).

To keep pace with the rapidly mutating viruses that thrive in challenging environments, eukaryotes have evolved novel defense strategies (Lauring et al., 2013). The potent RNAi pathway is exceptionally dynamic, and the general principles are universal, with some exceptions. Furthermore, with the development of highly complex organisms, interferon (IFN)-based regulation and immunoglobulin systems were established to combat infections (TenOever, 2016).

The coevolution of defense and counter-defense mechanisms between the host and pathogens has led to the significant diversification in the early established prototype genes in the RNAi pathway. For example, prokaryotes possess an independent yet functionally analogous defense mechanism akin to the RNAi pathway seen in eukaryotes. Nonetheless, the homologous counterparts of the genes responsible for eukaryotic RNAi are often involved in other diverse processes in prokaryotes. As chordates have evolved IFN and immunoglobulin systems, where the virus cell entry is receptor mediated, widespread protection is conferred in the organisms with higher complexity. Therefore, in higher eukaryotes, the RNAi pathway is primarily utilized in the developmental processes and differential regulation of genes responsible for various cellular processes. The eukaryotic RNAi machinery seems to have assembled from diverse building blocks from archaea, bacteria, and viruses. During the eukaryotic evolution, the duplication event of the paralogous forms of the three key proteins: Ago, RNA-dependent RNA polymerase (RdRP), and Dicer (Dcr), subsequently led to the significant diversification of the RNAi pathway across each class of eukaryotes.

In the RNAi, small RNAs (sRNAs) regulate gene expression within the host system and play a significant role in development, disease, genome organization, and antiviral defenses. The pathway is initiated by cleaving long dsRNA or precursor-miRNAs (pre-miRNAs) with the stem-loop structure into small interfering RNAs (siRNAs) and microRNAs (miRNAs), respectively. While siRNA is almost always made up of perfect complementary double-stranded RNA, miRNA may possess one or more mismatches in the complementary strands. Furthermore, the RNA-induced silencing complex (RISC) is directed by siRNAs or miRNAs to cleave the cognate mRNA or inhibit its translation (Hannon, 2002; Wilson and Doudna, 2013).

This review highlights the current understanding of Dicer and its domains derived from a series of structural and functional studies carried out in the last 20 years.

## The Evolution of the RNAi Pathway

Multiple pieces of evidence suggest that the core components of RNAi are evolutionarily conserved, indicating the siRNA-based mechanism was already present in the last eukaryotic common ancestor (LECA) and the miRNA biogenesis pathway emerged before the divergence of plants and mammals (Shabalina and Koonin, 2008). Subsequently, the frequent duplication events in multiple eukaryotic evolutionary periods led to the formation of paralogs in the genes involved in orchestrating the RNAi. Simultaneously, several unicellular eukaryotic microbes, such as *Plasmodium falciparum*, *Saccharomyces cerevisiae*, and *Leishmania major*, have evolved as RNAi-deficient organisms where other regulatory defense systems have been established (Shabalina and Koonin, 2008). Remarkably, the restoration of RNAi in *S. cerevisiae* by introducing AGO1 and DCR1 from the close member *S. castellii* did not demonstrate any developmental or growth defects; however, the expression of AGO1 and DCR1 affected the endemic killer virus system, suggesting that the RNAi and killer virus systems were incompatible. The net selective advantage of killer hosts over RNAi in *S. cerevisiae* suggests that the absence of RNAi machinery in several unicellular organisms transpired through the selection pressure (Drinnenberg et al., 2011).

## The Discovery of Dicer

**FIGURE 1:**
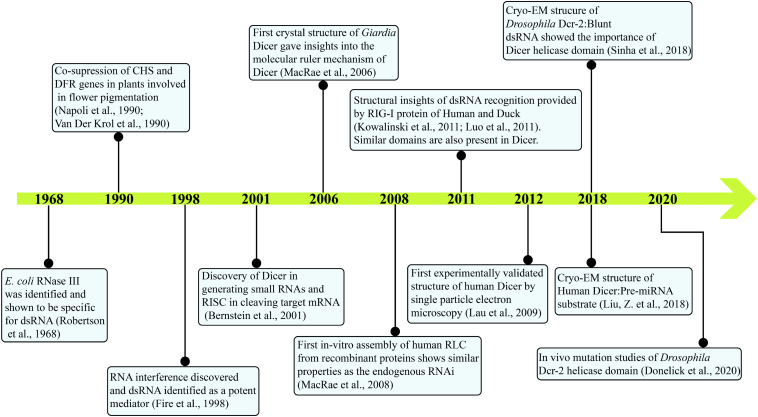
Major events in the timeline of Dicer research. Current knowledge on Dicer is attained by research from several groups and is annotated. With the identification of *E. coli* RNase III in 1968 to the present day, cryo-EM structures of *Hs*Dcr and *Dm*Dcr-2 depict the intricate work carried out through decades.

Initial indications of a specific enzyme involved in the post-transcriptional gene silencing (PTGS) pathway as a response to viral and transgene infection in plants were stemmed from the presence of 25 nt RNA product (Hamilton and Baulcombe, 1999). Subsequently, in the laboratory of Prof. Hannon, then a Ph.D. scholar, Emily Bernstein identified Dicer as a gene responsible for affecting the upstream part of RISC assembly, which recognizes the trigger dsRNA in a nucleotide sequence independent manner (Bernstein et al., 2001). In this classical study, they have shown the presence of a single class II gene, Drosha, and two class III genes CG4792 and CG6493 in *Drosophila* S2 cells. As Drosha was earlier known to be involved in the ribosomal RNA (rRNA) processing, both class III genes were considered as putative initiators of the RNAi pathway and were respectively named as Dcr-1 and Dcr-2. The study further showed that Dcr-1 diced the dsRNA precursors into ∼22 nt small dsRNAs and unraveled the central role of Dicer in initiating the RNAi pathway. The biochemical activity assay of small dsRNA generation by Dicer, using *Drosophila* embryo extract also showed greater cleavage efficiency for longer dsRNA precursors (400–500 bp) compared with relatively smaller dsRNA precursors (200–300 bp) (Bernstein et al., 2001). It was further shown that the *Dm*Dcr-1 is engaged in producing miRNAs, whereas *Dm*Dcr-2 is involved in producing endogenous siRNAs (endo-siRNAs) and exogenously triggered RNAi (exo-siRNAs) as well as they both work at the distinct steps for the siRNA-dependent RISC assembly (Lee et al., 2004). A time line of major discoveries leading to the current understanding of Dicer biology is given in Figure 1.

## The Origin of Dicer

**FIGURE 2:**
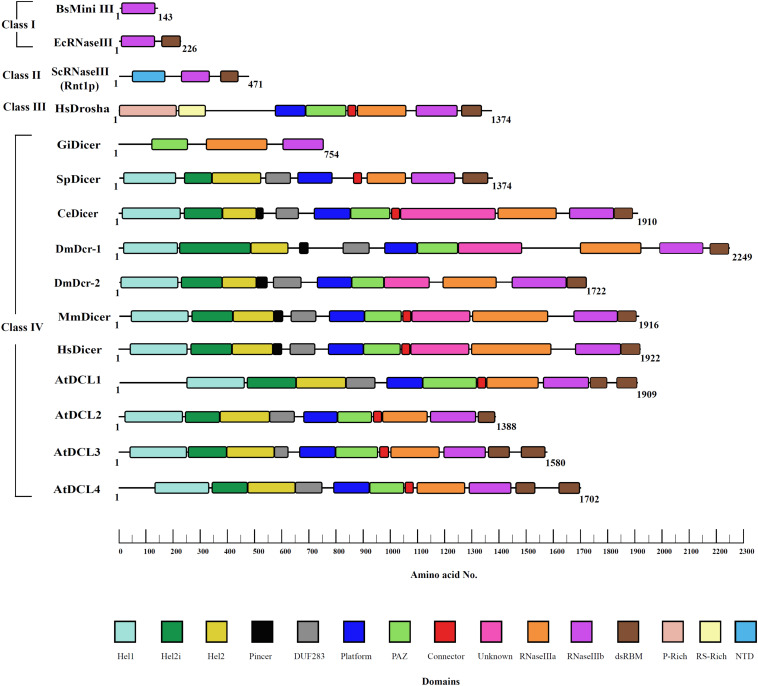
RNase III family of proteins. RNase III proteins are classified into four distinct classes based on the domain architectural complexity. Bacteria like *E. coli* and fungi like *S. cerevisiae* contain the simplest RNase III enzyme that belongs to class I and class II, respectively. Class III consists of Drosha with P-rich and RS-rich domains unique to this class of proteins mediating cleavage of primary-miRNA transcripts as seen in humans and *Drosophila*. Class IV consists of an evolved Dicer family of proteins. Although belonging to fungi, *S. pombe* has more evolved Dicer as compared to that of other fungal species. However, it lacks the PAZ domain essential for RNA recognition. The innovative domain shuffling of various domains in Dicer is evolved to respond to the needs of the species and changing environmental conditions. The domain boundaries were defined by Phyre-based homology modeling, conserved sequences of domains, and available X-ray, NMR, and cryo-EM structures.

Dicer is an endonuclease and belongs to the ribonuclease III (RNase III) family of enzymes, which is further divided into evolutionarily conserved four major classes as shown in Figure 2.

The class I RNase III enzyme possesses an Mg^2+^ dependent RNase III domain, along with a double-stranded RNA binding domain (dsRBD), and is widely present in bacteria, bacteriophage, and few lower eukaryotes. In this class, the RNase III domain undergoes homodimerization to bring two functional RNase III domains together for the cleavage of dsRNA. Interestingly, sweet potato chlorotic stunt virus (SPCSV) encodes a class I RNase III enzyme that mediates silencing of the RNAi pathway by cleavage of active sRNAs into inactive fragments. Furthermore, it promotes coinfection with sweet potato feathery mottle virus (SPFMV), causing severe growth defects in sweet potato plants, presenting unusual viral synergism for plant infection (Cruzado et al., 2009). Class I RNase III enzymes also include Mini-III in *Bacillus subtilis* with a single RNase III that catalyzes 23S ribosomal RNA maturation (Aguado and TenOever, 2018). Also, class II, class III, and class IV RNase III enzymes have been evolved exclusively in eukaryotes by selecting novel domains to carry out specialized functions. In class II RNase III enzymes, the introduction of an N-terminal domain, which imparts homodimerization, can be observed. The class II enzymes are involved primarily in processing precursors of ribosomal RNA, small nuclear RNA (snRNA), and small nucleolar RNA (snoRNA). The more evolved class III enzymes have an additional RNase III domain, along with the dsRBD, which enables its function as a monomer. The enzyme family consisting of Drosha proteins, which process precursor miRNA in the nucleus of eukaryotic cells, is a classic example of the class III RNase family of enzymes. The final class IV has a conserved helicase domain, similar to that present in the RIG-I-like receptor (RLR) family of proteins, in addition to the aforementioned domains that discriminate it from other RNase III classes. Dicer is classified as a class IV RNase III enzyme, and, in humans, it contains a helicase domain, two RNase III domains, two dsRBDs, and a Piwi/Ago/Zwille (PAZ) domains. Interestingly, despite having its own dsRBDs, *Homo sapiens Dicer* (*Hs*Dcr) Dicer depends on one or more auxiliary proteins that contain more than one dsRBD for the initiation of the RNAi as well as loading of a correct strand during the pre-RISC formation (Chendrimada et al., 2005).

The absence of Dicer in prokaryotes and its central role in eukaryotic gene regulation suggest that Dicer is an early eukaryotic innovation (Cerutti and Casas-Mollano, 2006). The earliest stages of Dicer evolution probably occurred through the shuffling and fusion of helicase, RNase III, and PAZ domains, which ensured the multifaceted role currently played by Dicer (Shabalina and Koonin, 2008). From several studies performed so far, it is clear that almost all eukaryotes possess one or more Dicer, with a series of functional domains that recognize and cleave dsRNA in a coordinated manner by partnering with RNA-binding proteins. While a single Dicer gene is found in vertebrates and nematodes, the presence of two and four Dicer genes, respectively in insects and plants, has been ascribed to the functional distinction of miRNA biogenesis and antiviral-based siRNA biogenesis (Margis et al., 2006). The presence of multiple Dicer genes in insects and plants reflects the diverse environmental background and threats that prevailed during evolution. Following the evolution of multicellularity, the Dicer family was evolved and also mirrors the independent evolution of Dicer in animals, plants, and fungi (Mukherjee et al., 2013).

## Disparity in the Number of Dicers Across Species

Although the basic domain architecture of Dicer is highly conserved across eukaryotes, many species utilize more than one Dicer for functional advantages.

The unicellular eukaryote *Tetrahymena thermophila (Tt)* encodes three Dicers annotated as Dicer-like protein 1 (DCL1), producing ∼27–30 nt sRNAs, DCR1, and/or DCR2 involved in the production of ∼23–24 nt sRNAs and, also, with distinct expression patterns (Mochizuki and Gorovsky, 2005; Lee and Collins, 2006). Cell processes based on RNAi are best understood with ciliates, such as *Tetrahymena* and *Paramecium* as the model systems where sRNAs distinguish genomes of generative and somatic cells (Nekrasova and Potekhin, 2018).

The filamentous fungus *N. crassa* possesses two functionally redundant Dicers, DCL1, and DCL2 that are responsible for the quelling (the transgene-induced gene silencing) as well as dsRNA processing into 25 nt long siRNA products (Catalanotto et al., 2004).

In plants, four different Dicers emerged by gene duplication occasions and were present after the divergence of animals and plants and before the separation of monocotyledonous and dicotyledonous plants (Margis et al., 2006). Plants lack an adaptive immune system but possess immune capabilities in the form of proteins called “pathogen recognition receptors” (PRRs) that recognizes different patterns of pathogens and places the plant in defense mode, referred to as “pattern-triggered immunity” (PTI) (Zipfel, 2014). The receptor and cell wall independent entry of viruses makes it possible for the siRNA to track the virus movement and combat infections (Heinlein, 2015). Thus, in plants, RNAi is the key regulator of infections. The four DCLs in *Arabidopsis thaliana* associate with five dsRNA Binding Proteins (dsRBPs), DRB1 to DRB5 (Hiraguri et al., 2005). Under normal developmental conditions, the *At*DCL1:DRB1 complex is involved in the miRNA biogenesis, generating 21 bp small RNA, leading to transcript cleavage; however, upon induction of biotic or abiotic stress, *At*DCL1 interacts specifically with DRB2 to mediate translational inhibition (Reis et al., 2016). *At*DCL4:DRB4 complex generates 21 bp siRNA to regulate trans-acting siRNA and viral siRNA pathways (Deleris et al., 2006; Nakazawa et al., 2007). DRB4 also interacts with DRB7.2 and DRB7.1 to regulate endogenous inverted-repeat (endo-IR) triggered siRNA response by sequestration of dsRNA precursors processed by *At*DCL3 (Montavon et al., 2017; Tschopp et al., 2017). Similarly, the *At*DCL3:DRB3 complex mediates the RNA-directed DNA methylation (RdDM) pathway by generating 24 bp siRNAs, whereas, *At*DCL2 is involved in transgene silencing by the production of 22 bp siRNAs (Mlotshwa et al., 2008; Raja et al., 2014). Furthermore, DRB2, DRB3, and DRB5 coordinate the formation of the cytoplasmic puncta that inhibit viral replication (Raja et al., 2014).

As mentioned earlier, *Drosophila* RNAi is mediated by two Dicer paralogs with multiple partnering dsRBPs. The Dcr-1:Loquacious (Loqs-PA or PB) complex was shown to be involved in miRNA biogenesis for generating ∼22 nt miRNAs. Similarly, the tandem dsRNA-binding domains (R2) in heterodimeric complex with Dcr-2 (D2), i.e., R2D2, and Loqs-PD complexes with Dcr2, *viz.*, Dcr2:R2D2 and Dcr2:LoqsPD are involved in mediating the siRNA biogenesis, generating 21 nt siRNAs (Liu et al., 2003; Saito et al., 2005). While the Loqs-PD:Dcr2 complex ensures that an upstream event of cleavage of endogenous-long dsRNA into small siRNA is carried out effectively, the association of R2D2 with *Dm*Dcr-2 is crucial to load the antisense siRNA on Ago2 (Tomari et al., 2004; Liu et al., 2006; Miyoshi et al., 2010).

In other organisms, including humans and worms, a single Dicer is involved in generating both siRNAs and miRNAs by association with one or more regulatory dsRBPs.

In humans, two dsRBPs, PKR activator (PACT), and HIV-1 TAR RNA-binding protein (TRBP), dsRBPs in humans, interact with Dicer to induce distinctive effects on substrate selection and cleavage (Chendrimada et al., 2005; Haase et al., 2005). TRBP and PACT possess two N-terminal dsRBDs accountable for dsRNA-binding activity, whereas the third dsRBD mediates interaction with Dicer (Lee et al., 2006; Yamashita et al., 2011). By coupling with Dicer, dsRBPs likewise affect siRNA/siRNA^∗^ and miRNA/miRNA^∗^ duplex loading onto Ago. For example, TRBP is one of the principal components of ribonucleoprotein effector complexes: RISC in humans (Chendrimada et al., 2005; Haase et al., 2005; MacRae et al., 2008). Furthermore, it was shown that TRBP enhances the Dicer cleavage activity by interacting with the Dicer helicase domain and by stabilizing the Dicer:dsRNA complexes (Chakravarthy et al., 2010).

In *C. elegans*, DCR-1 is involved in the biogenesis of both miRNA and siRNA, where RDE-4 (dsRBP) and RDE-1 (Ago) assist DCR-1 to generate ∼22 bp sRNAs (Tabara et al., 2002; Parker et al., 2006, 2008; Welker et al., 2007). By investigating the role of RDE-4 domains through mutation studies, it was shown that the linker region and dsRBD2 were necessary and sufficient for DCR-1 interaction and, subsequently, for the activation of the RNAi pathway in *C. elegans* (Blanchard et al., 2011).

## Insights From *in vitro* Dicer Cleavage Assays

Insights into unique dsRNA substrate preferences and distinct length specific generation of sRNAs by Dicer have been obtained by the *in vitro* Dicer cleavage assays from several research groups in various organisms. For *A. thaliana*, crude extracts of seedlings and radiolabeled dsRNA were used to show that DCL3 prefers shorter dsRNA substrates (<50 nt), and DCL4 prefers longer dsRNA substrates (>50 nt) for cleavage to generate 24 and 21 bp sRNAs, respectively (Nagano et al., 2014). Interestingly, the addition of recombinant DRB4 protein rescued the DCL4-mediated cleavage in the *drb4* mutant plants, implying the interdependence of partner dsRBP and corresponding Dicer (Fukudome et al., 2011). DCL3 was further shown to cleave 1 nt and 2 nt 3′ overhang dsRNA products through the 5′ end counting rule as *Hs*Dcr (Nagano et al., 2014). At the same time, the association of DCL1 with DRB1 or DRB2 yielded a 21 nt siRNA product (Kurihara et al., 2006).

Similarly, the crude extracts of *N. crassa* were used for the cleavage assay and displayed the production of 23 nt sRNA products, specifically by *Nc*DCL2, as dicing activity was identified in the Δ*dcl1* mutant strain but not in the Δ*dcl2* mutant (Tabara et al., 2020). Nonetheless, in addition to 23 nt products, the presence of 18 and 28 nt small dsRNA fragments suggested that the *Nc*DCL2 may have been an early homolog of Dicer, which led to the evolution of plant DCL3 and DCL4 (Tabara et al., 2020). Likewise, the crude extracts of *Drosophila* S2 cells are used widely for *in vitro* assays. Interestingly, these Dicer activation assays revealed that the B2 protein of the Flock House Virus (FHV) inhibits Dicer activity in a dose-dependent manner and acts as an antiviral suppressor of RNAi (Aliyari et al., 2008; Yang and Li, 2011). Furthermore, the Dicer activity of *Nc*DCL2, *Dm*Dcr-2, and *At*DCL4 was also shown to be ATP dependent (Fukunaga et al., 2014; Nagano et al., 2014; Tabara et al., 2020).

*Giardia intestinalis* Dicer (*Gi*Dcr) cleaves dsRNA in the presence of Mg^2+^ to produce 25–27 nt sRNAs that mediate gene silencing (MacRae et al., 2006). In *C. elegans*, *in vitro* Dicer cleavage was established, using embryo extract to understand the individual steps of RNAi and showed the requirement of ATP hydrolysis for processing dsRNA precursors and not miRNA precursors (Ketting et al., 2001). The cleavage assay of *Ce*Dcr-1 showed the key role of dsRBP RDE-4, as Dcr-1 was unable to cleave dsRNA in *rde-4* mutants, and cleavage activity was recovered after the addition of recombinant RDE-4 in the Dicer assay (Parker et al., 2006). *Hs*Dcr can tolerate remarkable structural variation in pre-miRNAs. A large-scale dicing assay performed by Feng et al. (2012) surveyed nearly 161 classes of pre-miRNAs, and surprisingly, the *Hs*Dcr cleaved most of the pre-miRNAs with different cleavage efficiencies. The cleavage assays of *Hs*Dcr showed significant cleavage preferences to 2 nt 3′ overhang, double helical nature in the stem region, and relatively, the large terminal loop (≥10) in the pre-miRNA substrates.

## Functional Insights Through Structural Outlook

The early efforts to unravel the structure-based functional understanding of the role of Dicer in the initiation of RNAi came from the crystal structure of *Gi*Dcr solved at 3.3 Å resolution. Although *Gi*Dcr lacks the important N-terminal helicase domain, the structure explained the length specificity of sRNA products, employing the “molecular ruler” that emerged from the spatial separation of the tandem RNase III and PAZ domains (MacRae et al., 2006). Subsequent crystal structure of mouse Dicer RNase IIIb and dsRBD domains in the presence and absence of Cd^2+^ revealed symmetric homodimer as seen in bacterial RNase III (Du et al., 2008). The study further showed that a conserved lysine (K1790) is vital to Dicer’s functioning as its mutation-impaired dsRNA cleavage ability (Du et al., 2008).

**FIGURE 3:**
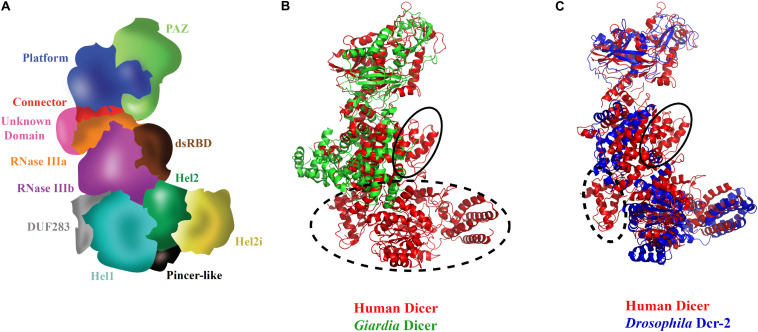
Three-dimensional view of Dicer and its domains. **(A)** Scheme of *Hs*Dcr domain arrangements prepared based on the cryo-EM structure (Liu et al., 2018). The unique structural arrangement of domains makes it possible for processing several kinds of dsRNA substrates. **(B)** Overlay of *Hs*Dcr (PDB code: 5ZAK) and *Gi*Dcr (PDB code: 2QVW). The PAZ and RNase III domains in *Gi*Dcr are necessary and sufficient for dsRNA cleavage. However, *Hs*Dcr possesses an additional helicase domain which is important in recognition of different terminal loop lengths of pre-miRNAs for efficient cleavage. The dashed circle in the overlay shows the absence of DUF283 and helicase domains in *Gi*Dcr and the solid circle indicates the absence of the dsRBD domain in *Gi*Dcr. **(C)** Overlay of cryo-EM structures of *Hs*Dcr (PDB code: 5ZAK) and *Dm*Dcr-2 (PDB code: 6BUA) indicates overall conservation of domains in the three-dimensional space in diverse eukaryotes. The helicase subdomain Hel1 (DExD/H-box domain) which is important for ATP hydrolysis is conserved in both *Hs*Dcr and *Dm*Dcr-2. However, the *Hs*Dcr helicase domain functions in an ATP-independent manner, whereas the ATP hydrolysis by *Dm*Dcr-2 Hel1 domain allows it to recognize blunt-end dsRNA for viral defense. The dashed circle in the overlay shows the absence of cryo-EM density for DUF283 in *Dm*Dcr-2 and the solid circle shows the absence of cryo-EM density for the dsRBD domain in *Dm*Dcr-2, indicating their dynamic nature in comparison with the *Hs*Dcr. The PDB files were superimposed and the images were created using Pymol.

Initial structural studies, using negative-stain EM, 3D reconstruction of *Hs*Dcr revealed the overall L-shaped architecture as shown in Figure 3A (Lau et al., 2009). Furthermore, cryo-EM studies on *Hs*Dcr and *Dm*Dcr-2 displayed the overall domain arrangement with the Hel1 subdomain at the junction of the short and long arms of Dicer interacting with RNase IIIb and Domain of unknown function 283 (DUF 283) domains. Hel2i subdomain is at the tip of the short arm of Dicer, and the PAZ domain is at the tip of the long arm of Dicer. The overlay of *Hs*Dcr with *Gi*Dcr and *Dm*Dcr-2, as shown in Figures 3B,C, reveals the conservation of domains at the structural level. It was identified through the 2D classification of cryo-EM reconstructions that the helicase domain was more flexible than the rigid core of Dicer (Taylor et al., 2013). The helicase domain of Dicer plays regulatory roles and exhibits conformational dynamics of the Hel2 and Hel2i subdomains around Hel1, as shown in *Hs*Dcr, similar to that revealed in duck RIG-I (Kowalinski et al., 2011; Liu et al., 2018). The crystal structure of *Hs*Dcr Hel2i [partner-binding domain (PBD) domain] demonstrated that it makes a strong interaction, mediated through a series of hydrophobic contacts with the third dsRBD of TRBP (TRBPD3) (Wilson et al., 2015). The conserved interface residues of TRBP and PACT portray that the two dsRBPs bind Dicer, using a similar D3 domain yet in a mutually exclusive manner. Importantly, the complex structure of Hel2i:TRBPD3 elucidates that the two N-terminal dsRBDs of TRBP, which are responsible for the recognition of triggered dsRNA, are free to adopt any orientation as they are connected by two long flexible linkers made up of ∼63 amino acids.

Dicer performs canonical and non-canonical cleavage of dsRNA-generating heterogeneous sRNAs that lead to off-target effects. Through *in vitro* reconstituted systems, it has been established that the Dicer is cleaved by “3′ end counting rule,” as shown in *Gi*Dcr, and “5′ phosphate end counting rule,” as shown in *Hs*Dcr (MacRae et al., 2007; Park et al., 2011). However, the cleavage in the case of pre-miRNAs with loops and bulges is not understood completely yet. “Loop counting rule” was proposed for the generation of homogeneous sRNA products *in vivo* from pre-miRNAs in which the cleavage is determined by the distance between the canonical cleavage site and loop structure (Gu et al., 2012).

The Dcr1 of budding yeast *Kluyveromyces polysporus* displays a non-canonical mode of dsRNA cleavage. The unique mechanism of initiating dsRNA cleavage from within rather than the termini of dsRNA substrates presents a paradigm shift from the regular Dicer molecular ruler mechanism. The distance between the two active sites in RNase III domains of ∼20 Å apart, as shown in *Aquifex aeolicus* RNase III (AaRNase III) and *Gi*Dcr, is also conserved in *K. polysporus* Dcr1 (Gan et al., 2006; MacRae et al., 2006; Weinberg et al., 2011). The 23 nt specific product length generated by budding yeast Dicer is mostly through the adjacent positioning of the two RNase III dimers on dsRNA (Weinberg et al., 2011). The study also showed termini-independent cleavage of dsRNA substrates by Dicer, unlike the canonical Dicer that requires free helical regions of dsRNA substrates for PAZ and helicase domain recognition (Liu et al., 2018; Sinha et al., 2018).

Recently derived cryo-EM structures of *Hs*Dcr and *Dm*Dcr-2 have opened a platform for understanding the mechanistic roles of various Dicer domains and also set a new avenue for the regulatory role of the conserved helicase domain of Dicer that is unique to the class IV RNase III family (Liu et al., 2018; Sinha et al., 2018). The overall L-shaped framework of Dicer, along with the precise position of Dicer domains arranged in a peculiar topology, was initially built in the *Hs*Dcr:TRBP complex. In this structure, the helicase domain forms the base, whereas the platform-PAZ domain appears at the top, and the RNase IIIa–RNase IIIb domains form the intramolecular dimer in the center (Lau et al., 2009; Liu et al., 2018; Sinha et al., 2018). A comprehensive list of known structures of Dicer and relevant domains, along with the corresponding PDB IDs, is listed in Table 1.

**TABLE 1:** RNase III enzymes and Dicer full length and domain structures in the complex without or with dsRNA/substrates along with their PDB ID.

PDB code	Domain/protein/complex	Sources
5ZAK	Human Dicer:TRBP	Liu et al., 2018
5ZAL	Human Dicer:TRBP:pre-let-7 (class I)	Liu et al., 2018
5ZAM	Human Dicer:TRBP:pre-let-7 (class II)	Liu et al., 2018
4WYQ	Human Dicer PBD:3rd dsRBD of TRBP	Wilson et al., 2015
2EB1	Human RNase IIIb domain	Takeshita et al., 2007
5B16	Human Drosha:C-terminal tail of DGCR8	Kwon et al., 2016
4GL2	Human MDA-5:dsRNA	Wu et al., 2013
2YKG	Human RIG-I	Luo et al., 2011
5E3H	Human RIG-I	Jiang et al., 2011
4NHA	Human Dicer platform:PAZ:connector helix:6 mer dsRNA	Tian et al., 2014
4NGD	Human Dicer platform:PAZ:connector helix:12 mer dsRNA	Tian et al., 2014
3C4B	Mouse RNase IIIb domain:dsRBD domain of Dicer	Du et al., 2008
3C4T	Mouse RNase IIIb domain:dsRBD domain of Dicer + Cd^+2^	Du et al., 2008
4A36	Duck RIG-I helicase:19 mer dsRNA	Kowalinski et al., 2011
4A2P	Duck RIG-I helicase	Kowalinski et al., 2011
6BUA	*Drosophila* Dcr-2	Sinha et al., 2018
6BU9	*Drosophila* Dcr-2^*RIII*^:52 BLT dsRNA:ATP-γS	Sinha et al., 2018
2KOU	*Arabidopsis* DCL4 DUF283	Qin et al., 2010
2LRS	*Arabidopsis* 2nd dsRBD of DCL1	Burdisso et al., 2012
2FFL	*Giardia* Dicer	MacRae et al., 2006
2QVW	*Giardia* Dicer	MacRae and Doudna, 2007
3RV0	*Kluyveromyces* Dcr1ΔCdsRBD2	Weinberg et al., 2011
3RV1	*Kluyveromyces* N-terminal:RNase III^*E*224*Q*^ domains	Weinberg et al., 2011
2L6M	*Schizosaccharomyces* dsRBD of Dicer	Barraud et al., 2011
5T16	*Saccharomyces* RNase III(Rnt1p)	Iii et al., 2017
2EZ6	*Aquifex* RNase III (D44N):dsRNA	Gan et al., 2006
2NUG	*Aquifex* RNase III:RNA 7	Gan et al., 2008
2NUF	*Aquifex* RNase III:RNA 7:RNA 8:Mg^2+^	Gan et al., 2008
2NUE	*Aquifex* RNase III:RNA 9:Mg^2+^	Gan et al., 2008
1R6Z	*Drosophila* Ago2 PAZ domain with MBP	Song et al., 2003
1R4K	*Drosophila* Ago1 PAZ domain	Yan et al., 2003

The complex structure of *Hs*Dcr PBD domain with TRBPD3 yielded, for the first time, mapped the interaction region of the functional complex between a Dicer and dsRBP. The complex structure provided the basis for the organization of TRBP and *Hs*Dcr through a set of well-defined hydrophobic interactions. Furthermore, through the comparison with RIG-I helicase, the authors showed that the Dicer helicase domain independently interacts with dsRNA and TRBPD3, and these sites do not induce steric hindrance. Additionally, the stability of Dicer was found to be enhanced as TRBPD3 masked the hydrophobic patch on the Dicer PBD domain. With the previous structures of TRBP dsRBD1 (TRBPD1) and TRBP dsRBD2 (TRBPD2), the authors modeled an overall spatial arrangement of *Hs*Dcr:TRBP complex to process miRNAs efficiently (Yamashita et al., 2011). Subsequently, it was shown that the TRBPD1 and TRBPD2 help in recognizing the thermodynamically more stable end of dsRNA and help loading the less stable end onto the Ago (Wilson et al., 2015).

The long-standing question of how the *Hs*Dcr:TRBP complex processes different pre-miRNA precursors into products of specific and identical features was partially answered by the recent cryo-EM structures of the complex, along with the pre-miRNA (Liu et al., 2018). Significant conformational heterogeneity in the pre-miRNAs warrants that the Dicer:dsRBP complex must recognize the stable stem-duplex structures in the pre-miRNA. The cryo-EM structure demonstrated the swivel motion of the *Hs*Dicer PBD:TRBPD3 region, which allows the terminal loop of pre-let-7 miRNA to reorient in the cleavage-competent state, thus, validating the idea of the “loop counting model” proposed in 2012 (Gu et al., 2012; Wilson et al., 2015). Hence, the helicase domain makes the Dicer unique in its ability to process RNA in comparison with the bacterial RNase III proteins (Liu et al., 2018).

In the subsequent sections, we will elaborate on some of the key domains of Dicer that are essential for catalytic activity.

## dsRNA Recognition Mechanism Through the Helicase Domain

A well-known class of mammalian innate immune response proteins is DExD/H-box RNA helicases that are classified in the super family 2 group of helicases (Gorbalenya et al., 1989). Vertebrates rely on RLRs, laboratory of genetics and physiology (LGP2), melanoma differentiation associated gene-5 (MDA5), and retinoic acid inducible gene I (RIG-I) for antiviral defense mechanisms, whereas invertebrates rely on Dicer. As RLRs recognize and suppress viruses independent of downstream antiviral signaling pathways, MDA5 was generated from the Dicer helicase domain, with the incorporation of two sequential Caspase activation and recruitment domains (CARDs) and RLR specific C-terminal domain (CTD). Further gene duplication of MDA5 led to RIG-I leading to the evolution of the IFN-based system in mammals. However, studies have shown that MDA5 and RIG-I are developed independently by domain embedding rather than a simple gene duplication episode (Sarkar et al., 2008). IFN system is absent in invertebrates and plants that function via RNAi for antiviral suppressing (Veen et al., 2018).

**FIGURE 4:**
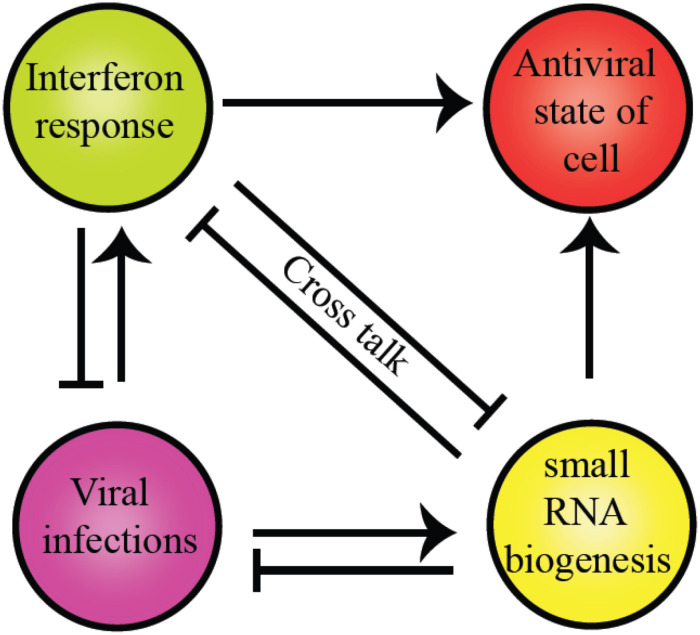
Crosstalk between RLR family of proteins and RNAi pathway components present in humans. The commonality between the two is the evolutionarily conserved helicase domain. Both pathways establish an antiviral state within the cells to maintain cell homeostasis. However, the presence of a particular response inhibits other pathways to supersede the antiviral response.

The intricate relationship between the RNAi pathway and IFN response in mammals establishes an antiviral state in the cell, as shown in Figure 4. Several miRNA genes regulate the levels of pro-inflammatory cytokines during viral infections. Type I IFNs regulate miRNAs in the post-transcriptional manner (Watson et al., 2019). In mammals, to execute the best defense strategies during infections by bacteria, fungi, viruses, and other parasites, the host defense pathways such as the classical innate immune responses and the RNAi interact. The potential of helicases to couple ATP hydrolysis with dsRNA binding induces conformational changes to perform dual functions like sensors and effectors. Dicer, the core component of the RNAi pathway, is a classic example of a helicase executing dual functions as a viral sensor and antiviral effector in amplifying the ‘signal’ for the downstream complex, RISC.

**FIGURE 5:**
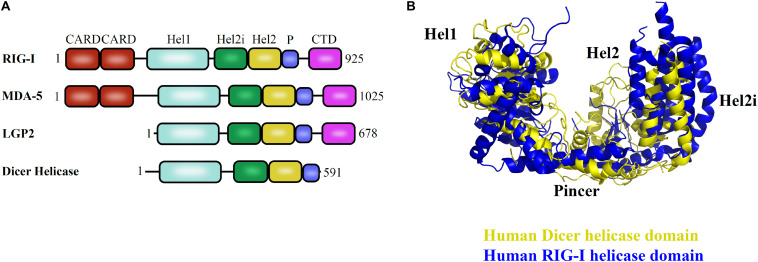
Helicase domain of RLR family of proteins and Dicer in humans. **(A)** Conserved domain architecture of proteins functioning in different pathways. **(B)** Overlay of *Hs*Dcr helicase (PDB code: 5ZAL) and human RIG-I helicase domains (PDB code: 2YKG) suggests the overall conservation in the three-dimensional orientation of subdomains. The positioning of Hel2 and Hel2i subdomains around the Hel1 domain forms a groove that is essential for interaction with the terminal loop of pre-miRNA as shown in *Hs*Dcr and to mediate interaction with blunt dsRNA precursor as demonstrated in *Dm*Dcr-2 (Liu et al., 2018; Sinha et al., 2018).

The helicase domain of Dicer is highly homologous to RIG-I/MDA5/LGP2, with three subdomains: Hel1, Hel2i, and Hel2 and overall conservation of 3D architecture as depicted in Figures 5A,B (Ahmad and Hur, 2015). Despite multiple studies performed so far, many aspects of the helicase domain of Dicer are still elusive in understanding its biological role. The *Hs*Dcr helicase domain was shown to weaken the dsRNA cleavage efficiency by 65-fold when compared with the deleted construct (Ma et al., 2008). Therefore, the helicase domain might serve as the structural switch regulating the Dicer cleavage activity. The *Hs*Dcr helicase domain is necessary and sufficient for the interaction with TRBP (Ma et al., 2008). The Dicer cleavage activity is fueled by ATP hydrolysis in *Drosophila*, *C. elegans*, *S. pombe*, and most invertebrates (Ketting et al., 2001; Singh et al., 2020). However, the Dicer belonging to the human and other vertebrates function independently of ATP (Zhang et al., 2002).

## PAZ Domain: Specific Recognition of Two 3’ Terminal Nucleotides

**FIGURE 6:**
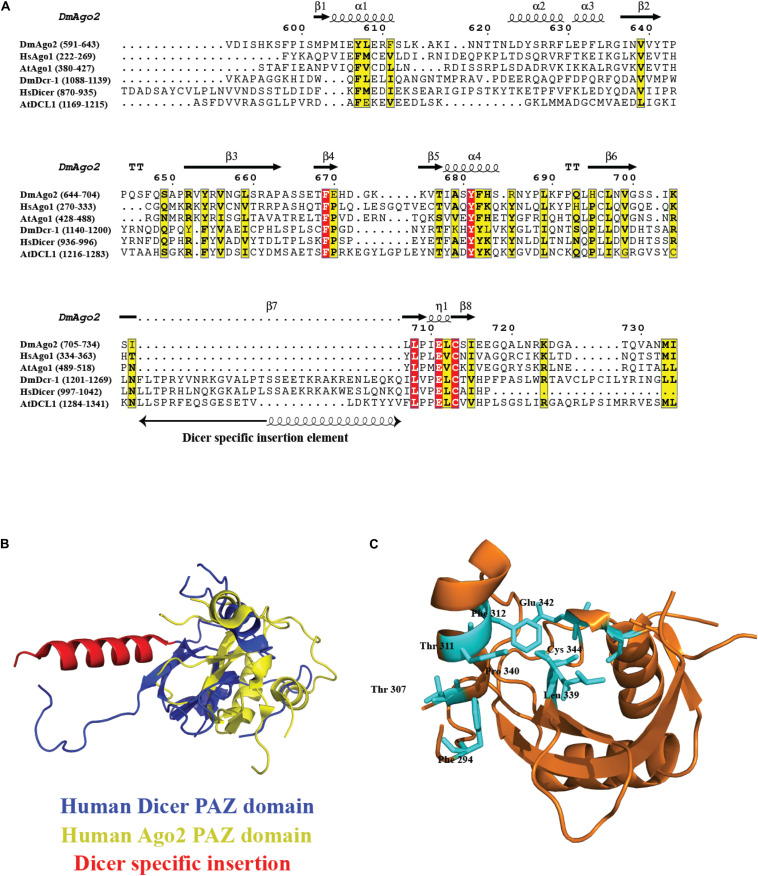
Conservation of PAZ domain in the Ago and Dicer family. **(A)** Sequence alignment of the PAZ domain present in different organisms shows several key residues involved in RNA recognition are conserved. In the case of PAZ in Dicer proteins, there is an extra insertion of a helix. **(B)** Overlay of *Hs*Dcr PAZ domain (PDB code: 4NGD) and *Hs*Ago2 PAZ domain (PDB code: 4W5O). The overall fold is conserved with the additional helix protruding out of the main fold. **(C)** The ribbon diagram of the PAZ domain from *Hs*Ago2 shows invariant residues highlighted in cyan present in all the members.

Piwi/Ago/Zwille domain plays an important role in the biology of the RNA-silencing mechanism. Ago proteins and Dicer contain PAZ domain, a variant of oligonucleotide/oligosaccharide binding(OB)-fold involved in single-stranded nucleic acid binding (Murzin, 1993). The dsRNA-binding pocket in PAZ for 2nt 3′ overhang dsRNA is highly conserved and was proposed for *Dm*Ago2 PAZ domain, *Hs*Ago1, and other PAZ domains identified to date through structure-based studies, sequence conservation mapping, and mutagenesis experiments (Lingel et al., 2003; Song et al., 2003; Yan et al., 2003). The residues for RNA binding are conserved across PAZ-containing Ago and Dicer homologs, as shown in the sequence conservation analysis in Figure 6A, with Dicer-specific insertion of an additional α-helix, as seen in the overlay of *Hs*Dcr PAZ domain (PDB code: 4NGD) and *Hs*Ago2 PAZ domain (PDB code: 4W5O) in Figure 6B, with the overall fold being conserved. Furthermore, Figure 6C shows the invariant residues conserved in all the PAZ domain-containing proteins, with the *Hs*Ago2 PAZ domain as an example. Exclusive distribution of PAZ in Dicer and Ago proteins indicates their roles in siRNA/miRNA loading and siRNA/miRNA guide strand selection, respectively.

The inherent dsRNA measuring capability of Dicer depends on the PAZ domain through its specific RNA binding and its structural placement. The 2nt 3′ overhang of the dsRNA is recognized by the PAZ domain and is identical to the Ago PAZ domain. The very first Dicer endoribonuclease activity models were proposed in which the distance between the PAZ domain and the RNase III catalytic core determined the length of sRNAs (Zhang et al., 2004). The first structural evidence of how Dicer acts as a molecular scale to cleave the dsRNA substrates was shown for *Gi*Dcr (MacRae et al., 2006). It performs the catalysis function similarly, using two-metal ions as the other bacterial RNase III proteins. The ∼65 Å distance between the PAZ domain 3′ overhang binding site and the catalytic sites in RNase III domains is equivalent to ∼25 bp RNA product generated by *Gi*Dcr (MacRae et al., 2006). The *Gi*Dcr mutated in the PAZ domain cleaved dsRNA at variable positions, generating different lengths of sRNA products. Furthermore, electrostatic interactions with the dsRNA backbone by surface residues connecting the PAZ and RNase III domains also play a subtle role in positioning dsRNA (MacRae et al., 2007). For *Hs*Dcr, RNase III domains and PAZ domain are separated by a distance of ∼55 Å, generating 21 bp RNA product (Lau et al., 2012). The spatial separation is attributed to the presence of additional accessory domains, such as a platform and dsRBD, in eukaryotic Dicers that locate the PAZ domain at a variable distance from two RNase III domains, resulting in sRNA products of different lengths. *S. pombe* lacks the PAZ domain, suggesting an alternative mechanism of dsRNA recognition that is yet to be unraveled (Colmenares et al., 2007).

## RNA Cleavage Mechanism Through RNase III Domain

Over the evolutionary time, RNase III nucleases have formed the base of the RNA metabolic machinery. The *E. coli* RNase III was first discovered by Robertson et al. (1968). All the members of the RNase III family of proteins are defined by their ability to cleave dsRNA and generate 2nt 3′ overhang as the signature on shorter RNA fragments of 18–24 bp. The structural simplicity of bacterial RNase III makes them an ideal candidate to study the mechanistic details of this family of proteins. The selective recognition and cleavage of dsRNA by the RNase III are conserved and follow a pattern of a coordinated reaction. The basic mechanism of catalysis of the RNase III domain is universally applied to all the proteins in the RNase III family. The simplistic model of prokaryotic RNase III provided insights into this process. Belonging to the superfamily of polynucleotidyl transferases, RNase III also uses metal ion-mediated hydrolysis reactions like the transposases, RNases, and DNases (Steiniger-White et al., 2004; Nowotny and Yang, 2006). Furthermore, the dimerization of the RNase III domains creates a catalytic center. Though each member of RNase III exhibits differing substrate specificity and generates distinct product length, superimposition of the RNase III structures revealed the conserved residue position-mediating hydrolysis.

The hallmark features of dsRNA in adopting the A-form of the double-stranded helix with thermodynamic stability obtained with metal ion interactions, along with its ability of 2′OH groups to give stable C_3_′-endo sugar conformation, make it specific for cleavage (Nicholson, 2014). The RNase III enzyme emerged in an early prokaryote and reached the eukaryote through endosymbiosis. Moreover, it was evolved by the acquisition of auxiliary domains to perform specialized functions. A special fold (∼150 aa), with a 10 aa consensus sequence to homodimerize and bind divalent metal ions to catalyze dsRNA, is adopted by the RNase III domain. The biochemical and structural studies of *Thermotoga maritima* RNase III and *Aa*RNase III–RNA complexes set forth the catalytic mechanism of dsRNA cleavage (Gan et al., 2008; Meng and Nicholson, 2008; Nicholson, 2014). Additional studies on *Aa*RNase III, *Gi*Dcr, and *Hs*Dcr showed two metal ions in each catalytic site. In *Gi*Dcr, the replacement of Mg^2+^ with Er^3+^ prevented catalysis. One Er^3+^ was coordinated with an acidic residue side chain cluster (D,D,E,E) and the other metal ion outside the cluster (MacRae et al., 2006).

**FIGURE 7:**
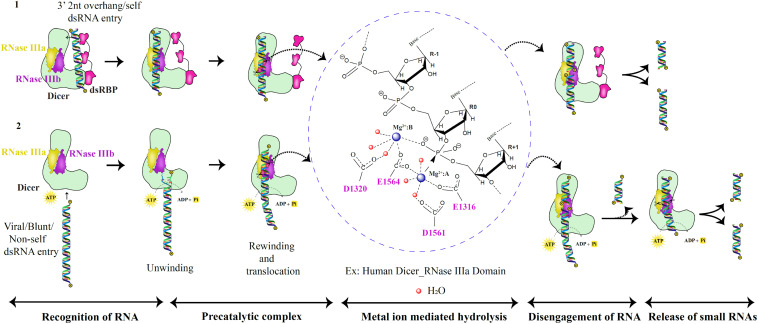
RNase III catalytic mechanism of the Dicer family of proteins. Based on the catalytic pathway proposed for prokaryotic RNase III enzymes, the eukaryotic dsRNA processing by Dicer is classified into five critical steps as shown above (Ji, 2006; Meng and Nicholson, 2008; Nicholson, 2014). In the first scenario, the PAZ domain recognizes dsRNA substrates with 2nt 3′ overhang. Besides, the dsRNA substrate is placed between the PAZ and RNase III domains to produce unique lengths of sRNAs via two catalytic metal ion mechanisms. In the second scenario, dsRNA with blunt termini are recognized by the helicase domain in an ATP-dependent manner.

“Single dsRNA processing center” was proposed for bacterial and *Hs*Dcr, with two RNA catalytic sites, generating two nucleotide 3’ overhang products (Zhang et al., 2004). The intramolecular dimerization of RNase III domains in Dicer mediates cleavage as opposed to bacterial intermolecular dimerization of RNase III. It was further shown that the mutation in the active site residues of the RNase IIIa domain did not affect the cleavage ability of the RNase IIIb domain of Dicer and vice versa (Zhang et al., 2004). Hence, the independent functioning and asymmetric placement of the catalytic regions of RNase IIIa and RNase IIIb domains mediate cleavage of 3′ hydroxyl and 5′ phosphate-bearing RNA strands, respectively (Zhang et al., 2004). The acidic residue cluster coordinated to Mg^2+^ ions associates with the water molecules in a series of proton transfers to mediate the hydrolysis reaction in the catalytic site. The metal ions play a vital role in the cleavage of RNA by appropriate positioning of the protein-RNA complex and placing the water molecules at the right distance from the oxygen atom of the phosphate group. Recent studies, using MD simulation, have shown that the OH^–^ group performs the nucleophilic attack rather than the water molecule in bimolecular S_*N*_2 reaction (Drusin et al., 2020). The mutated *Aa*RNase III (E40Q, D44N, D107N, and E110Q), with no metal ion, bound to RNA but lost the ability to cleave (Gan et al., 2008), thus presenting the metal ion as the conserved component of the dsRNA catalysis throughout the evolutionary period, as shown in Figure 7, in the metal ion hydrolysis step, with *Hs*Dcr RNase IIIa as an example. The 2 Å *Hs*Dcr RNase IIIb domain structure (PDB code: 2EB1) displayed similar characteristics to other the RNase III bacterial enzymes and the *Gi*Dcr RNase III domains. It had two Mg^2+^ ions per monomer in the catalytic site, where MgA ion serves as a catalyst and MgB ion essential for RNA binding activity, as shown in *Aa*RNase III and *Ec*RNase III (Takeshita et al., 2007).

Eukaryotic Dicers employ the same cleavage strategy that is established by the prokaryotic RNase III enzymes. However, the recognition of different dsRNA substrates by Dicer is possible by its PAZ and helicase domains that mediate two different mechanisms of dsRNA recognition as shown in Figure 7. The PAZ domain present in the protein family of Dicer and Ago is unusual in its ability to identify 2nt 3′ dsRNA overhang as seen in *Gi*Dcr and *Hs*Dcr (MacRae et al., 2006; Tian et al., 2014; Liu et al., 2018). The efficiency of dsRNA recognition and cleavage by Dicer is enhanced with the assistance of dsRBPs. Examples include *Hs*Dcr:TRBP complex, *At*DCL4:DRB4 complex, *Dm*Dcr2:LoqsPD complex, and *Ce*Dcr:RDE-4 complex (Tabara et al., 2002; Fukudome et al., 2011; Liu et al., 2018; Sinha et al., 2018). Helicase domain present in the RLR family of proteins and Dicer recognizes dsRNA in an ATP-dependent manner. One such example is *Dm*Dcr-2 that cleaves blunt dsRNA substrates (Sinha et al., 2015). Furthermore, dsRNA is unwound and threads through the helicase domain and rewinds and translocates to place the RNA at the PAZ domain. The cleavage of the first ∼15 bp dsRNA is performed to generate the 2nt 3′ overhang dsRNA for PAZ domain recognition from blunt dsRNA precursors as recently shown for *Dm*Dcr-2 (Donelick et al., 2020). Future work on Dicers from different organisms might reveal other mechanisms of dsRNA recognition and cleavage apart from the two known mechanisms to date.

## Involvement of Dicer in siRNA Loading Onto RISC

**FIGURE 8:**
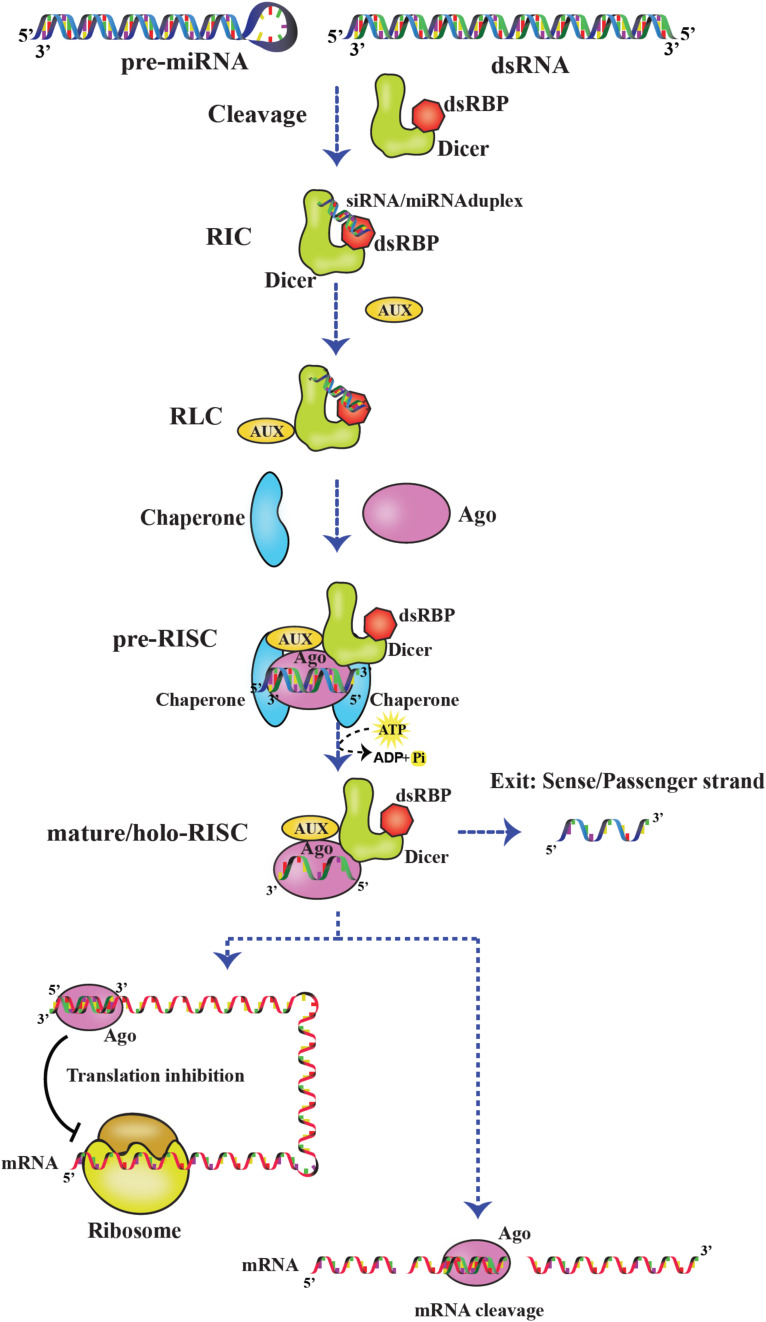
RNA-induced silencing complex (RISC). Dicer:dsRBP complex cleaves pre-miRNA and dsRNA precursors to generate sRNAs. Later, the sRNAs are loaded on the Dicer:dsRBP complex to form RIC. Further, interaction with other auxiliary proteins (AUX) forms RLC. Ago interacts with RLC to form pre-RISC where the small RNA is loaded on Ago. The passenger strand exits and the guide strand is retained on Ago to form mature/holo-RISC. Based on the base pairing of the guide strand and target mRNA in the seed region, the mRNA is translationally inhibited or cleaved.

The RISC assembly facilitates the recruitment of sRNAs that are transferred by Dicer and used for screening cognate mRNA in highly complex and coordinated steps as depicted Figure 8. RISC initiation complex (RIC) consists of Dicer and dsRBP with the sRNA for cognate mRNA silencing. Dicer acts as the primary sensor for siRNA selection by recognizing 2 nt 3′ overhang and thermodynamically unstable ends of the siRNA. Thus, by selective binding of siRNAs, Dicer serves as a gatekeeping function to distinguish effective and non-effective siRNAs. However, the thermodynamic asymmetric sensing of siRNA is primarily carried out by R2D2 in *Dm*Dcr–2:R2D2 complex in which R2D2 positions siRNA by binding thermodynamically a more stable end and recognizing the 5′ phosphate so that Dcr-2 binds to the thermodynamically less stable end, preceding the pre-RISC formation (Tomari et al., 2004). Furthermore, NMR-based approaches showed that the tandem dsRBDs of Loqs-PD are involved in asymmetric sensing of siRNA through sliding and associating with the less stable end of RNA and interacting with Dcr-2 forming RIC (Tants et al., 2017).

Moreover, RIC is associated with other auxiliary proteins to form RISC-loading complex (RLC), and sRNAs are loaded onto Ago by RLC-forming pre-RISC. For example, *Hs*Dcr:TRBP complex loading miRNAs onto *Hs*Ago2 and *Dm*Dcr–2:R2D2 complex loading siRNAs onto *Dm*Ago2 (Kim et al., 2006; Wilson et al., 2015). Previous studies have shown the importance of chaperones-regulating pre-RISC complex formation, mediated by ATP hydrolysis. In humans and *Drosophila*, Hsp90/Hsc70 chaperone machinery is involved in sRNA duplex loading onto Ago (Iwasaki et al., 2015; Naruse et al., 2018). As soon as the siRNA/miRNA duplex is loaded, the passenger strand is cleaved and/or removed by Ago. The remaining guide strand on Ago forms mature/holo-RISC-facilitating cleavage or translational suppression of cognate mRNAs at their 3′ untranslated regions by complementary base pairing. The recognition of the guide strand is carried out by different domains of AGO. The middle (MID) domain interacts with the 5′ end of small RNA, and the PAZ domain interacts with the 2 nt 3′ overhang of small RNA in a conserved pocket. Furthermore, the PIWI domain cleaves the mRNA (Greenidge et al., 2019).

At the same time, in humans, Dicer ensures accurate loading of a guide strand on Ago, and the asymmetric sensing ability of Dicer is further enhanced by TRBP/PACT (Noland and Doudna, 2013; Wilson et al., 2015).

Dicer:dsRBP complex has distinct roles in generating small RNA duplexes from longer dsRNA precursors and loading of sRNAs onto Ago (Lee et al., 2004). Eventually, the Dicer:dsRBP complex interacts directly with Ago. A vertebrate-specific minimal and unique amino acid- binding motif in RNase IIIa domain of Dicer, interacting with the Ago family of proteins, was reported that was absent in invertebrates (Sasaki and Shimizu, 2007). Subsequently, Ago carries out the second proofreading mechanism for the appropriate guide strand loading onto the RISC complex.

## Association of RdRP and Dicer in the RNAi Pathway

From unicellular organisms, such as *Giardia* to multicellular complex organisms, such as humans and plants, RdRP is present in one or more copies. In all organisms, RdRP generates dsRNA precursors from a single stranded RNA (ssRNA) template (Maida and Masutomi, 2011).

The *Tt*Dcr-2’s activity in producing 23–24 nt sRNAs is enhanced by its association with RNA-dependent RNA polymerase Rdr1. *Tt*Dcr-2 has less cleavage specificity due to the lack of PAZ, dsRBD, and DUF283 domains that aid in dsRNA precursor selection (Lee and Collins, 2007). Hence, to limit the activity on Rdr1 dsRNA substrates, Dcr-2 efficiently cleaves dsRNA precursors with the Rdr1 initiation site (Lee and Collins, 2007). In *S. pombe*, a single copy of Rdp1, Dcr1, and Ago1 performs heterochromatin silencing mediated by RNAi (Sigova et al., 2004). However, as of today, no evidence exists that shows the direct association of RdRP Rdp1, and Dcr1 in *S. pombe*. The transcriptional gene silencing in *A. thaliana* is mediated by RNA-directed DNA methylation (RdDM), in partnership with RNA polymerase IV (Pol IV), to produce ssRNA from the DNA template that is converted by RDR2 into dsRNAs and is subsequently channeled into DCL3 to generate 24 nt siRNAs loaded onto Ago4 (Herr et al., 2005; Singh et al., 2019). Human telomerase reverse transcriptase (TERT) with RdRP activity is involved in *de novo* production of siRNA precursors, characterized by 5′-triphosphate termini of siRNAs in a Dicer-dependent manner (Maida et al., 2016).

## Role of Dicer in Genome Integrity

The pivotal role of Dicer is portrayed during the meiotic maturation, where the regulated use of stored mRNAs is achieved. A study in 2007 uncovered the novel role of RNAi in mammalian meiosis, where robust Dicer expression was seen in oocytes, and early embryos of mice, where the deletion of Dicer was embryonic lethal. It was further found out that the Dicer-deficient mouse oocytes were defective in the spindle organization and chromosome segregation during the metaphase-I or metaphase-II of meiosis-I and meiosis-II, respectively (Murchison et al., 2007). The balance between the siRNA and miRNA biogenesis in organisms, possessing a single Dicer gene, necessitated the evolution of altered Dicer isoforms. Early studies from the group of Petr Svoboda showed the presence of mouse oocyte-specific form of Dicer annotated as Dicer^*O*^, with truncated N-terminal domain that produces endo-siRNAs from long siRNA precursors, whereas, in full-length Dicer in somatic cells, Dicer^*S*^ processes pre-miRNAs (Flemr et al., 2013). During the germline development, the Dicer^*O*^ regulates the production of endo-siRNAs in mice, whereas, in organisms lacking Dicer^*O*^, the PIWI-associated RNA (pi-RNA) pathway assumes the central role (Flemr et al., 2013).

## RNAi Independent Interaction Network of Dicer

Cumulative evidence of extensive research indicates that, along with the production of small regulatory RNAs in the RNAi pathway, Dicer is also involved in various cellular processes like apoptotic DNA degradation, inflammatory responses, and heterochromatin remodeling (Kurzynska-Kokorniak et al., 2015). The cellular levels of Dicer need to be maintained as even small deviation leads to pathological conditions such as neurodegenerative disorders and carcinogenesis in humans. It has been shown that many cellular gene regulatory machineries influence Dicer gene expression, translation, and enzymatic activity. For example, *Hs*Dcr gene expression is positively regulated by the SRY-box transcription factor 4 (SOX4) that decides the cell fate (Jafarnejad et al., 2013). Post-translational modifications of Dicer also regulate its activity like phosphorylation by an Extracellular signal-Regulated Kinase (ERK), causing nuclear translocation of Dicer in humans, mice, and worms (Drake et al., 2014). Dicer interacts with ribosomal DNA (rDNA) repeats, thereby enhancing the stability of these regions reported in mammals, yeast, and flies (Cam et al., 2005; Peng and Karpen, 2007; Sinkkonen et al., 2010). During double-strand breaks (DSBs) in the genome, Dicer is involved in cell response pathways-generating sRNAs, called “DSB-induced sRNAs” (diRNAs) from sequences in the vicinity of DSB sites as shown in animals and plants (Francia et al., 2012; Wei et al., 2012). Furthermore, diRNAs facilitate the recruitment of protein complexes to the repair site, thus maintaining the genome integrity and enhancing the survival ability of the cell. Some of the RNA viruses interact directly with Dicer through their proteins, like the core protein of hepatitis C virus (HCV) and the Trans-Activator of Transcription (TAT) in the Human immunodeficiency viruses type I (HIV-1), to suppress RNAi (Bennasser and Jeang, 2006; Chen et al., 2008). Dicer is also shown to interact with membrane-associated proteins. *Hs*Dcr interacts with the 63 kDa cytoskeleton-linking membrane protein (CLIMP-63) to regulate Dicer levels (Pépin et al., 2012). *Hs*Dcr dsRBD domain was shown to interact in a dsRNA independent manner, with 5-lipooxygenase (5LO) involved in catalysis of inflammatory mediators, and regulate each other’s activity (Dincbas-Renqvist et al., 2009). The transactivation domain of tumor protein p63 (TAp63) that belongs to the p53 family enhances Dicer gene expression during the DNA damage response (Su et al., 2010). It is well established that caspases promote apoptosis of the cell in diverse eukaryotes. Caspase cell death protein 3 (CED-3) in *C. elegans* cleaves Dcr-1 to generate the deoxyribonuclease C-terminal fragment that cleaves DNA-causing chromosomal degradation, a hallmark of apoptosis (Nakagawa et al., 2010). The repertoire of Dicer-interacting proteins has broadened with increasing shreds of evidence, suggesting its multifaceted roles.

## Regulation of Dicer

The regulation of Dicer is shown to occur at the cellular level by several factors. For example, *Hs*Dcr is upregulated by IFN-γ and downregulated by Reactive Oxygen Species (ROS), activation of Rat Sarcoma (RAS) oncogene, and type I IFNs (Wiesen and Tomasi, 2009). The RAS-ERK pathway and the RNAi pathway, mediated by Dicer, are evolutionarily conserved and play crucial roles in several developmental events and diverse cellular processes. The ERK-Dicer network regulates several processes in different cellular contexts. Additionally, ERK and Dicer expressions during germline development are well conserved from worms to humans (Knight and Bass, 2001; Murchison et al., 2007). In animals and worms, the nuclear translocation of Dicer is regulated by phosphorylation of two conserved residues in the RNase IIIb and dsRBD domains, mediated by ERK (Drake et al., 2014). Hematopoietic cells, such as B cells, are highly sensitive to loss of Dicer and quickly go through apoptosis, which implies Dicer as a potential therapeutic target for controlling certain hematopoietic malignancies like the B cell lymphoma (Adams and Eischen, 2014). Along with the cellular energy sensors like AMP-activated protein kinase (AMPK) and mechanistic target of rapamycin (mTOR), Dicer also plays a critical role in energy homeostasis by generating miRNAs to maintain the nutrient status in mammals (Tong et al., 2020).

## Outstanding Questions With Exploratory Views

RNAi is a recent eukaryotic specific addition to the gene regulatory pathways, and Dicer is the central player in initiating the pathway through the biogenesis of precursor dsRNA and escorting the sRNAs for further processing through RISC. Dicer plays a pivotal role as a housekeeping protein in regulating normal cellular processes as well as in combating infections for the effective functioning of cells and, hence, the organism. The seeming heterogeneity in the function of Dicer despite possessing a high degree of functionally similar and conserved domains is quite perplexing. It seems that, on an evolutionary time scale, the adaptation of the multifaceted activities of Dicer is a recent response to assist the organism in adaptation and survival. Hence, studies of Dicer and Dicer-like proteins provide us an opportunity to study the evolution of versatility in the key pathways at the molecular level. Similarly, an in-depth understanding of the structural and structure-based biochemical and functional aspects of the RNAi pathway would eventually lead to harnessing the therapeutic potential of the RNAi pathway.

A study showed that Dcr-2, R2D2, and Ago2, proteins involved in the antiviral siRNA pathway of *Drosophila* were among the rapidly evolving 3% of *Drosophila* genes. However, the counterpart miRNA pathway involved in the housekeeping function of regulating gene expression did not show increased rates of amino acid changes over the evolutionary period (Obbard et al., 2006). A recent study on insect species has shown the structural variability of protein domains: helicase, RNase III, PAZ, and dsRBD domains of the core proteins, mediating key processes of RNAi (Arraes et al., 2020). The study provided clues for the application of RNAi technology in insect pest control. The evolutionary perspective of structural variability of RNAi pathway proteins in other organisms, such as humans, plants, and worms, will be of great advantage to explore RNAi in therapeutics much efficiently.

Some of the key questions that are still unanswered in the field are: why do plants and insects have multiple Dicers? And what is their origin? Are the mechanisms of dsRNA recognition and cleavage by Dicer in various organisms the same or different? How certain Dicers distinguish multiple dsRNA substrates, whereas some Dicers can only recognize a specific set of dsRNA precursors? How does the RNAi pathway coordinate spatially and temporally with the normal cellular machinery to regulate specific genes required for cellular homeostasis? Why has evolution laid alternative mechanisms of antiviral defense system despite the presence of Dicer and RNAi?

The study of Dicer-mediated RNAi initiation and effector complex is one of the most complex and challenging problems not only due to the large size of the molecules but also due to the significant conformational heterogeneity involved in the formation of such a complex. A series of high-resolution structures of the Dicer-induced ternary complex, capturing the intermediate states, would enhance our understanding of the mechanism of the activity of Dicer and the course of its evolution from lower eukaryotes to complex organisms.

## Author Contributions

SP and MD conceptualized the theme, surveys the literature, and wrote the review. Both authors contributed to the article and approved the submitted version.

## Conflict of Interest

The authors declare that the research was conducted in the absence of any commercial or financial relationships that could be construed as a potential conflict of interest.
